# Promotion of Hepatic Differentiation of Bone Marrow Mesenchymal Stem Cells on Decellularized Cell-Deposited Extracellular Matrix

**DOI:** 10.1155/2013/406871

**Published:** 2013-08-07

**Authors:** Hongliang He, Xiaozhen Liu, Liang Peng, Zhiliang Gao, Yun Ye, Yujie Su, Qiyi Zhao, Ke Wang, Yihong Gong, Fan He

**Affiliations:** ^1^Department of Infectious Diseases, Third Affiliated Hospital of Sun Yat-Sen University, 600 Tianhe Road, Guangzhou, Guangdong 510630, China; ^2^Key Laboratory of Tropical Disease Control, Ministry of Education, Sun Yat-Sen University, 600 Tianhe Road, Guangzhou, Guangdong 510630, China; ^3^Department of Biomedical Engineering, School of Engineering, Sun Yat-Sen University, No. 132, East Waihuan Road, Guangzhou Higher Education Mega Center, Guangzhou, Guangdong 510006, China; ^4^Guangdong Provincial Key Laboratory of Sensor Technology and Biomedical Instrument, Sun Yat-Sen University, No. 132, East Waihuan Road, Guangzhou Higher Education Mega Center, Guangzhou, Guangdong 510006, China; ^5^Orthopaedic Institute, Soochow University, 708 Renmin Road, Suzhou 215007, China; ^6^Department of Orthopaedics, The First Affiliated Hospital of Soochow University, 188 Shizi Street, Suzhou 215006, China

## Abstract

Interactions between stem cells and extracellular matrix (ECM) are requisite for inducing lineage-specific differentiation and maintaining biological functions of mesenchymal stem cells by providing a composite set of chemical and structural signals. Here we investigated if cell-deposited ECM mimicked *in vivo* liver's stem cell microenvironment and facilitated hepatogenic maturation. Decellularization process preserved the fibrillar microstructure and a mix of matrix proteins in cell-deposited ECM, such as type I collagen, type III collagen, fibronectin, and laminin that were identical to those found in native liver. Compared with the cells on tissue culture polystyrene (TCPS), bone marrow mesenchymal stem cells (BM-MSCs) cultured on cell-deposited ECM showed a spindle-like shape, a robust proliferative capacity, and a suppressed level of intracellular reactive oxygen species, accompanied with upregulation of two superoxide dismutases. Hepatocyte-like cells differentiated from BM-MSCs on ECM were determined with a more intensive staining of glycogen storage, an elevated level of urea biosynthesis, and higher expressions of hepatocyte-specific genes in contrast to those on TCPS. These results demonstrate that cell-deposited ECM can be an effective method to facilitate hepatic maturation of BM-MSCs and promote stem-cell-based liver regenerative medicine.

## 1. Introduction

Liver failure as a serious health problem currently only relies on clinical transplantation surgery [1]. Due to the high cost of surgical procedures, shortage of donors' liver grafts, and major immune rejections, cell-based liver tissue engineering instead sparked immense attraction in the treatment of end-stage liver cirrhosis and infections [2]. An amount of bioartificial liver support devices has been developed to prolong patients' lives that are mostly based on cell therapy using human [3] or animal hepatocytes [4]. Animal studies have shown that these devices temporarily improved or replaced liver functions such as urea, bile acids, and lipid metabolism [5]. However, this technology is limited because of the scarcity of human autologous hepatocytes and the risk of rejection to xenogenic cells [6]. 

Mesenchymal stem cells (MSCs) as a promising source for liver regenerative medicine, compared with mature hepatocytes, have advantages in various tissue sources, robust self-renewal potential, multilineage differentiation capacity, and immunological tolerance [7]. There is increasing evidence that MSCs have the potential to develop into hepatocyte-like cells *in vitro*, not only expressing hepatocyte-specific genes and proteins but also metabolizing urea and synthesizing albumin [8]. A previous clinical trial demonstrated that transplantation of bone marrow mesenchymal stem cells (BM-MSCs) improved short-term efficacy and long-term prognosis of liver failure patients [9]. However, cell transplantation therapy toward clinical applications remains challenging due to the poor efficiency of stem cell transdifferentiation and relatively lower biological functions in contrast to mature hepatocytes [10].

Extracellular matrix (ECM), providing biophysical and chemical signals, plays a pivotal role in stem cell adhesion, migration, proliferation, differentiation, and matrix remodeling [11]. Ouchi et al. demonstrated that coating of type I collagen and fibronectin enhanced the expression of liver-specific genes in primary hepatocytes [12]. ECM proteins, such as collagens and laminin, mixed with growth factors were potent to facilitate stem cells differentiating to hepatic lineage [13]. In addition, threedimensional (3D) bioscaffolds were developed to mimic *in vivo* extracellular matrix microenvironment to support cell survival and hepatic differentiation of MSCs and embryonic stem cells [14]. A recent report showed that decellularized biomatrix from liver organ largely preserved the structural and componential characteristics of the original tissue network and improved functions of adult hepatocytes [15]. Moreover, from the view of the interactions between cells and environment, cell-deposited ECM membrane preserved topographical structures and composition of various proteins to facilitate cells rapidly forming *in vivo* fibrillar adhesions, evidenced by links between *α*_5_ *β*_1_ integrin, paxillin, and fibronectin [16]. Numerous studies have been reported that ECM is essential to maintain differentiated phenotypes and liver-specific functions in primary hepatocytes [17]. Therefore, ECM is essential to construct *in vivo* stem cell microenvironment [18] and has potential to be utilized in stem cell *in vitro* expansion and differentiation [19]. 

In the current study, we obtain decellularized ECM deposited by BM-MSCs and hypothesize that cell-derived ECM provides natural stem cell extracellular microenvironment, improves MSC proliferation, and facilitates MSC differentiating to hepatocyte-like cells. Our long-term goal is to develop a suitable therapeutic strategy by utilizing decellularized ECM to produce sufficient functional hepatocytes for liver tissue engineering and treatment of chronic liver diseases.

## 2. Materials and Methods

### 2.1. Decellularization of Cell-Deposited ECM

Tissue culture polystyrene (TCPS) plates (Corning, Tewksbury, MA, USA) were firstly pretreated with 0.2% gelatin solution (Sigma-Aldrich, St. Louis, MO, USA) for 1 h at 37°C, followed by 1% glutaraldehyde (Sigma) and 1 M ethanolamine (Sigma) for 30 min separately at room temperature. BM-MSCs (Lonza Group Ltd., Basel, Switzerland) were seeded on pretreated plates in *α*-MEM medium (Thermo Fisher Scientific, Waltham, MA, USA) supplemented with 10% fetal bovine serum (FBS; Thermo Fisher Scientific), 100 U/mL penicillin, 100 *μ*g/mL streptomycin, and 0.25 *μ*g/mL fungizone (Invitrogen, Carlsbad, CA, USA). After reaching 90% confluence, 100 *μ*M of L-ascorbic acid phosphate (Sigma) was added, and cells were cultured for additional 8 days. To decellularize cell-deposited ECM, cells were removed by PBS supplemented with 0.5% Triton X-100 (Sigma) and 20 mM NH_4_OH (Sigma) for 5 min at 37°C, rinsed with PBS, and stored at 4°C for future use.

### 2.2. Scanning Electron Microscopy (SEM) of Cell-Deposited ECM

Decellularized cell-deposited ECM were fixed in 4% paraformaldehyde (Sigma) and dehydrated in a series of alcohol at increasing concentrations (50%, 75%, 80%, 95%, and 100% solution). The morphology of decellularized ECM was examined by a scanning electron microscope (SEM S-520; Hitachi High-Technologies, Tokyo, Japan).

### 2.3. Immunofluorescence Staining

ECM was fixed in ice cold methanol for 10 min, blocked in 1% BSA, and incubated in appropriately diluted primary antibodies: antitype I collagen, antitype III collagen, antifibronectin, antilaminin (Abcam, Cambridge, MA, USA) and antidecorin (Santa Cruz Biotechnology, Dallas, TX, USA). After three rinses with PBS, ECM was incubated with a secondary antibody (Alexa Fluor 488 donkey anti-mouse IgG [H + L] or Alexa Fluor 488 donkey anti-rabbit IgG [H + L]) (Invitrogen). The fluorescence images were obtained by an IX71 fluorescence microscope (Olympus Corporation, Tokyo, Japan) and processed with Image-ProPlus software (Media Cybernetics Inc, Rockville, MD, USA).

### 2.4. Cell Culture and Fluorescein Diacetate (FDA) Staining

 BM-MSCs were seeded in 24-well plates at a density of 1,000 cells/well at 37°C with 5% CO_2_ under two different conditions: TCPS and ECM. The medium was changed every other day. Cells were washed with PBS and then incubated in FDA (5 *μ*g/mL; Sigma) solution at 37°C for 10 min. After rinsing with PBS, fluorescent images were captured by an Olympus IX71 microscope and processed with Image-ProPlus software.

### 2.5. Cell Proliferation Assay

As described previously [20], BM-MSCs (*n* = 5) were lysed, and the amount of DNA was measured with Quant-iT PicoGreen dsDNA assay kit (Invitrogen) using a SynergyMx Multimode Reader (BioTek, Winooski, VT, USA) as described by the manufacturer.

### 2.6. Measurement of Intracellular Reactive Oxygen Species

Intracellular reactive oxygen species (ROS) generation was measured with 2′, 7′-dichlorofluorescein diacetate (DCFH-DA; Sigma). In brief, 2 × 10^5^ cells (*n* = 4) were incubated in 10 *μ*M of DCFH-DA for 20 min at 37°C. DCF fluorescence was measured by a BD dual laser FACS Calibur (BD Biosciences, San Jose, CA, USA) with 10,000 events collected for each sample, and data were analyzed with WinMDI (Windows Multiple Document Interface for Flow Cytometry) 2.9 software.

### 2.7. Surface Markers Characterized by Flow Cytometry

 Samples (*n* = 3) of each 3 × 10^5^ BM-MSCs were firstly incubated in PBS containing 0.1% ChromPure Human IgG whole molecule (Jackson ImmunoResearch Laboratories, West Grove, PA, USA) and 1% NaN_3_ then in appropriately diluted mouse monoclonal antibodies of CD34, CD45, CD90, and CD105 (Abcam). After washing with cold PBS, BM-MSCs were incubated with the secondary antibody (Alexa Fluor 488 donkey anti-mouse IgG [H + L]). Negative controls received equivalent amounts of isotype-matched antibodies (Abcam). Cells were analyzed on a BD dual laser FACS Calibur (BD Biosciences, San Jose, CA, USA) with 10,000 events collected for each sample, and data were analyzed with WinMDI 2.9 software.

### 2.8. Hepatic Differentiation of BM-MSCs

To induce hepatic differentiation, BM-MSCs cultured on TCPS and ECM were incubated in DMEM/F12 medium (Thermo Fisher Scientific) supplemented with 10% FBS, 100 U/mL penicillin, 100 *μ*g/mL streptomycin, 0.25 *μ*g/mL fungizone, 20 ng/mL of HGF (PeproTech Asia, Rehovot, Israel), and 10 ng/mL FGF-4 (PeproTech Asia) for 2 weeks. Thereafter, differentiation medium was changed to maturation medium (DMEM/F12 supplemented with 10% FBS, 20 ng/mL oncostatin M [sigma], 100 *μ*M dexamethasone [sigma], ITS Premix (BD Biosciences, San Jose, CA, USA), 100 U/mL penicillin, 100 *μ*g/mL streptomycin, and 0.25 *μ*g/mL fungizone) and maintained for another 2 weeks. Medium was collected and stored at −80°C for the measurement of urea concentration.

### 2.9. Periodic-Acid-Schiff (PAS) Staining for Glycogen

Differentiated cells on day 21 and day 28 were fixed in 4% paraformaldehyde and then incubated in 1% periodic acid solution (Sigma) for 5 min at room temperature. Followed by rinsing with PBS, cells were incubated in Schiff's reagent (Sigma) for 15 min. Images were captured by an Olympus IX71 microscope.

### 2.10. Evaluation of Urea Synthesis

The concentration of urea in culture medium was measured by a commercially available QuantiChrom urea assay kit (BioAssay Systems, Hayward, CA, USA) according to the manufacturer's instructions. The absorbance was measured by a SynergyMx Multimode Reader at 520 nm.

### 2.11. Real-Time Reverse Transcription-Polymerase Chain Reaction (Real-Time RT-PCR)

Total RNA was extracted from samples (*n* = 4) by TRIzol reagent (Invitrogen). For each sample, 1 *μ*g of total RNA was reverse transcribed by PrimeScript RT reagent kit as described by the manufacturer (TaKaRa, Mountain View, CA, USA). To quantify the mRNA, cDNA equivalent to 20 ng of total RNA was used for real-time PCR analysis with GoTaq qPCR Master Mix (Promega, Madison, WI, USA). Genes including CuZn superoxide dismutase (CuZn-SOD), Mn superoxide dismutase (Mn-SOD), albumin (ALB), tryptophan 2,3-dioxygenase (TDO2), cytochrome P450 7A1 (CYP7A1), cytochrome P450 3A4 (CYP3A4), cytokeratin 18 (CK18), and hepatocyte nuclear factor 4 alpha (HNF-4A) were detected. GAPDH was as an internal standard. The primer sequences are listed in Table 1. real-time PCR was performed by an ABI7500 Realtime PCR Detection (Applied Biosystems, Foster City, CA, USA) and calculated with computer software (Perkin-Elmer, Wellesley, MA, USA).

### 2.12. Statistical Analysis

All data are expressed as mean ± standard error (S.E.). Statistical differences between two groups were determined by one-way analysis of variance (ANOVA) followed by Student's unpaired *t*-test with SPSS software package (SPSS Inc, Chicago, IL, USA). Significance is indicated by a *P* value of <0.05.

## 3. Results

### 3.1. Characterization of Decellularized ECM

**Figure 1:**
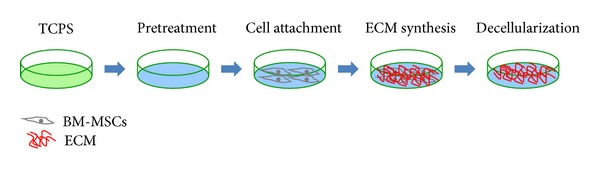
The protocol of preparing decellularized ECM. Conventional TCPS flasks were pretreated with gelatin, glutaraldehyde, and ethanolamine. L-ascorbic acid phosphate was supplemented to increase ECM production by BM-MSCs. ECM was decellularized by treating with Triton X-100 and NH_4_OH.

 The preparation process of decellularized ECM deposited by BM-MSCs was described in Figure 1. To optimize decellularized ECM for cell culture and differentiation, pretreatments with gelatin, glutaraldehyde, and ethanolamine were used to increase the adhesive strength between culture surface and ECM. L-ascorbic acid phosphate was added in culture medium to increase the generation of ECM and the treatment of Triton X-100 and NH_4_OH was used to remove original cells and cellular residues. 

**Figure 2:**
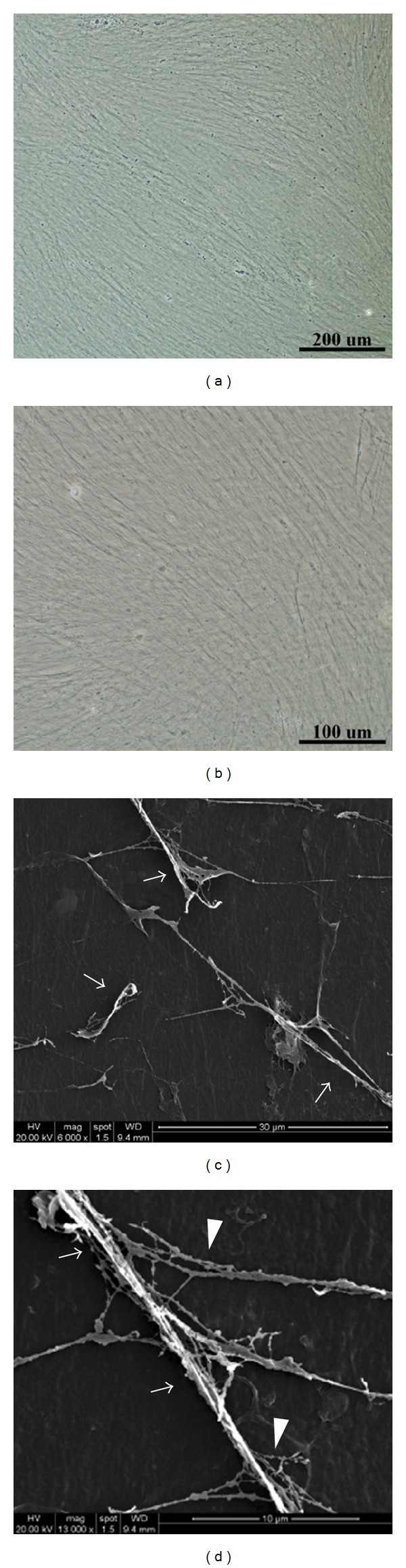
Characterization of decellularized cell-deposited ECM. The morphology of cell-deposited ECM showed fibrous structure under a light microscope ((a) scale bar = 200 *μ*m; (b) scale bar = 100 *μ*m), and SEM revealed fibrillar microstructure of ECM ((c) scale bar = 30 *μ*m; (d) scale bar = 10 *μ*m). A bundle of fibrillar collagen fibers (arrow) and beaded fibers (arrowhead) are observed.

Cell-deposited ECM after decellularization showed a fibrous structure (Figures 2(a)-2(b)) and the microstructure of fibrillar network was further observed via SEM (Figures 2(c)-2(d)). We found bundles of fibers (825.4 ± 114.3 nm in diameter), collagen fibrils (320.6 ± 49.5 nm in diameter), and beaded filaments that were possibly attached glycosaminoglycans. The gaps between fibers were left by decellularization of deposited cells, evidenced by a similar diameter of fibroblasts (4.7–11.7 *μ*m). 

**Figure 3:**
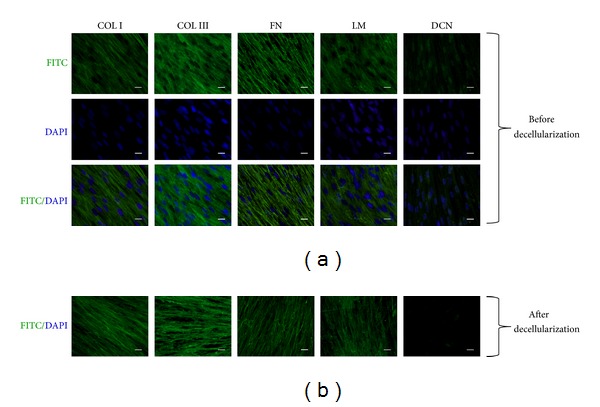
Detecting of multiply matrix proteins and cell nuclei in decellularized ECM before (a) and after (b) decellularization. The procedure of decellularization results in complete removal of original BM-MSCs in ECM deposition. Immunofluorescence staining of ECM retained type I collagen, type III collagen, fibronectin, and laminin except decorin. Scale bar = 20 *μ*m.

Immunofluorescence staining revealed that decellularization preserved most matrix proteins that were identical to native liver such as type I collagen, type III collagen, fibronectin, and laminin. However, decorin as a small proteoglycan that was expressed in cytoplasm or pericellular matrix was undetectable after decellularization. DAPI staining of ECM before and after decellularization confirmed the success of removing cellular residues (Figure 3). 

### 3.2. Cell Culture on Decellularized ECM

**Figure 4:**
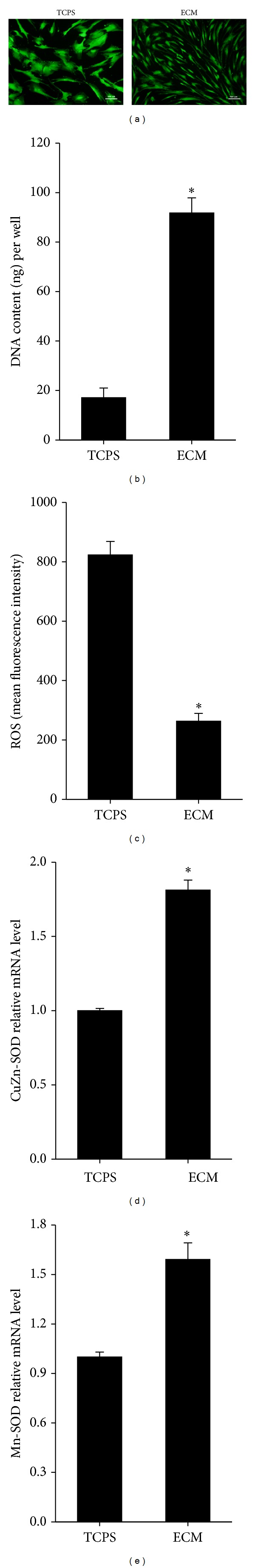
Cell culture of BMSCs on TCPS and cell-deposited ECM. Morphological changes and density of BMSCs were detected by FDA staining (a). ECM improved proliferation of BMSCs in 5-day culture (b). BMSCs on ECM showed a lower level of ROS than the cells on TCPS (c). mRNA levels of CuZn-SOD (d) and Mn-SOD (e) in the cultured cells were determined by real-time RT-PCR. Scale bar = 100 *μ*m. All values are mean ± S.E. of independent 4~5 experiments performed (proliferation assay  *n* = 5; ROS assay  *n* = 4; PCR  *n* = 4); *P < 0.05.

When cultured on ECM, BM-MSCs formed a small and spindle-like shape and maintained uniformly morphological appearance. In contrast, cells on TCPS showed a large and flattened morphology (Figure 4(a)). DNA content of BM-MSCs in 24-well plates was measured to evaluate proliferative activity. For cells cultured on ECM, DNA content is 5.4-fold as that of TCPS group (91.8 ± 6.1 ng/well versus 17.1 ± 3.9 ng/well) after 5-day culture (Figure 4(b)). With regard to intracellular ROS, cells on ECM showed a dramatically suppressed level in contrast to TCPS group (mean fluorescence intensity 263.2 ± 25.9 versus 823.4 ± 45.2), indicating that cell-deposited ECM was an effective culture system to reduce oxidative stress (Figure 4(c)). Moreover, mRNA expressions of CuZn-SOD (Figure 4(d)) and Mn-SOD (Figure 4(e)) were elevated by 81.2% ± 6.7% and 59.1% ± 10.1%, respectively, in the cells cultured on ECM than cells on TCPS. These results indicate that cell-deposited ECM abolishes redundant free radicals in BM-MSCs through superoxide dismutases pathway.

### 3.3. Immunophenotypes of BM-MSCs on ECM

**Figure 5:**
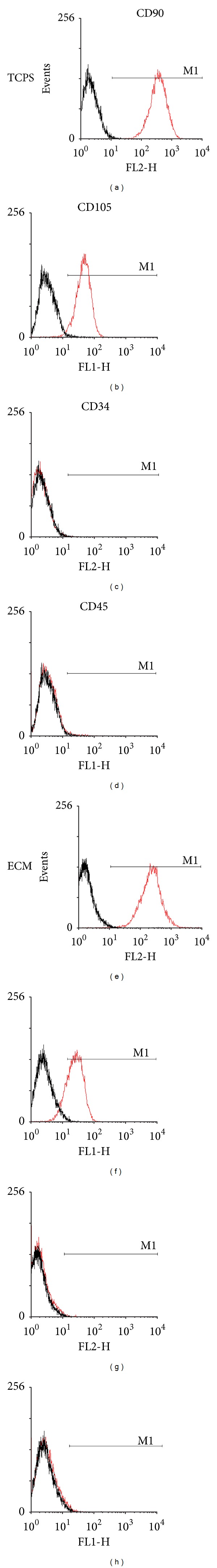
Immunophenotype analysis revealed that BMSCs cultured on TCPS and ECM were positive for CD90 and CD105 but negative for CD34 and CD45.

Flow cytometry analysis was performed to characterize the immunophenotypes of BM-MSCs cultured on TCPS or ECM. For standard MSC surface markers, the cells were strongly positive for CD90 (99.9% in TCPS versus 99.7% in ECM) and CD105 (96.5% in TCPS versus 81.5% in ECM), whereas the cells were negative for CD34 (0.5% in TCPS versus 0.8% in ECM) and CD45 (1.6% in TCPS versus 1.4% in ECM) (Figure 5). These data suggested that BM-MSCs expanded on decellularized ECM exhibited the same surface phenotypes as those cultured on TCPS. 

### 3.4. Effect of ECM on Liver-Specific Functions

**Figure 6:**
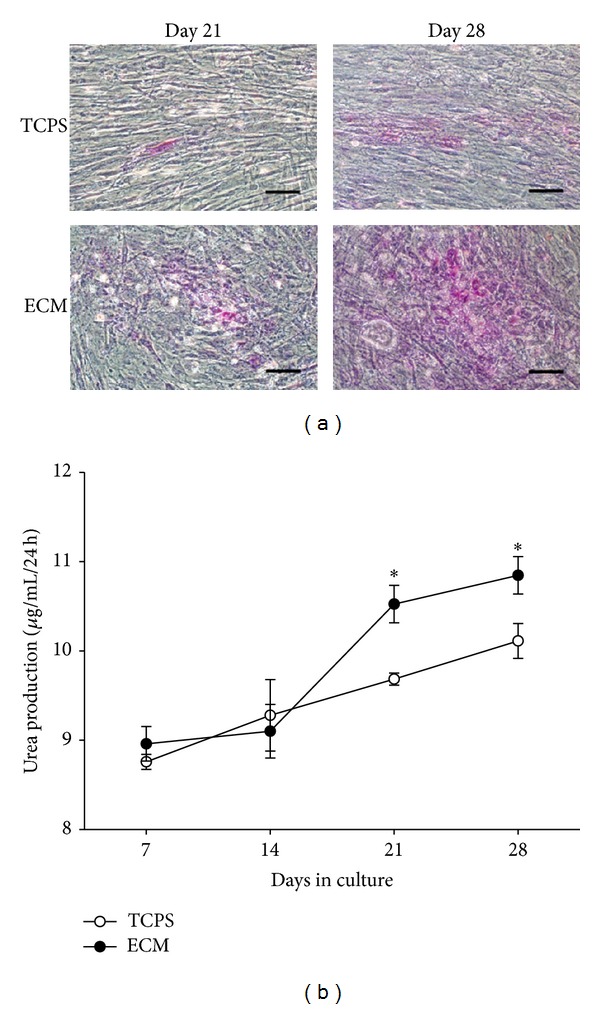
Cell-deposited ECM promoted hepatic differentiation of BMSCs. PAS staining of differentiated BMSCs on days 21 and 28 cultured on TCPS and decellularized ECM (a). Urea biosynthesis of differentiated BMSCs on TCPS and decellularized ECM on days 7, 14, 21, and 28, respectively, (b). Scale bar = 100 *μ*m. All values are mean ± S.E.; *P < 0.05.

BM-MSCs were induced to hepatogenesis in differentiation medium for 2 weeks and incubated in maturation medium for another 2 weeks. The morphology of BM-MSCs was changed from a spindly to round shape when cells were induced to differentiate on ECM. The results of PAS staining were positive in both TCPS and ECM groups on day 21, but the staining was significantly more intensive in ECM group compared with TCPS group on day 28 (Figure 6(a)). The result indicated that the ability of glycogen storage was enhanced in the differentiated cells on cell-deposited ECM.

The results of urea synthesis in differentiated BM-MSCs showed no significant difference on day 7 (8.8 ± 0.1 *μ*g/mL/24 h versus 9.0 ± 0.2 *μ*g/mL/24 h) or on day 14 (9.3 ± 0.4 *μ*g/mL/24 h versus 9.1 ± 0.3 *μ*g/mL/24 h) in TCPS and ECM groups. However, the urea concentration of differentiated BM-MSCs cultured on ECM was 8.7% higher than that of TCPS group on day 21 (10.5 ± 0.2 *μ*g/mL/24h versus 9.7 ± 0.1 *μ*g/mL/24 h, *P* < 0.05) and 7.3% higher on day 28 (10.9 ± 0.2 *μ*g/mL/24 h versus 10.2 ± 0.2 *μ*g/mL/24 h, *P* < 0.05) (Figure 6(b)). The data suggested that ECM improved the biological function of urea secretion in hepatocyte-like cells from BM-MSCs.

### 3.5. Expressions of Hepatocyte-Specific Genes in Differentiated BM-MSCs

**Figure 7:**
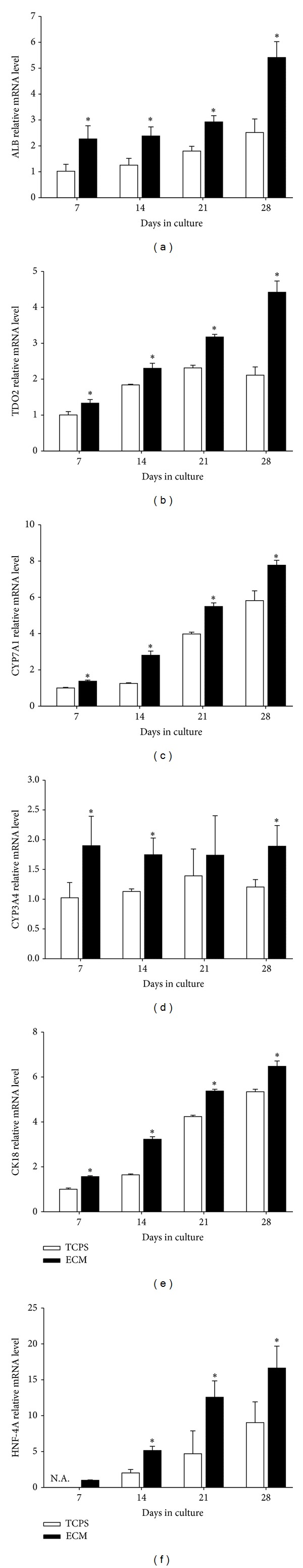
Expressions of hepatocyte-specific genes including ALB (a), TDO2 (b), CYP7A1 (c), CYP3A4 (d), CK18 (e), and HNF-4A (f) were examined by real-time RT-PCR on days 7, 14, 21, and 28, respectively. All values are mean ± S.E.; *P < 0.05; N.A.: not available.

Figure 7 shows relative mRNA expression of hepatocyte-specific genes, such as ALB, TDO2, CYP7A1, CYP3A4, CK18, and HNF-4A. Cell-deposited ECM significantly increased the expression of ALB compared with TCPS group by 89.9% on day 14 and by 114.9% on day 28 (Figure 7(a)). BM-MSCs cultured on ECM expressed a higher level of TDO2 than cells on TCPS (by 25.1% on day 14 and by 109.4% on day 28) (Figure 7(b)). mRNA of CYP7A1 was higher in the cells on ECM than TCPS group (by 123.6% on day 14 and by 33.5% on day 28) (Figure 7(c)). Similarly, cell-deposited ECM increased CYP3A4 mRNA by 54.8% on day 14 and by 57.0% on day 28 higher than TCPS (Figure 7(d)). With regard to genes of CK18 (Figure 7(e)) and HNF-4A (Figure 7(f)), ECM significantly upregulated mRNA levels in differentiated BM-MSCs compared with TCPS group (by 21.2% on day 28 of CK18 and by 84.1% on day 28 of HNF-4A).

## 4. Discussion

In the current study, we used decellularized ECM deposited by BM-MSCs as cell culture substrate to mimic *in vivo* stem cell microenvironment and examined the effects of cell-deposited ECM on cell proliferation, expressions of surface markers, stress of intracellular ROS, and hepatic lineage differentiation. Our data showed that the process of decellularization preserved the structure and most matrix components of ECM. In contrast to conventional TCPS monolayer culture system, BM-MSCs expanded on ECM showed similar expressions of stem cell surface markers, significantly increased cell proliferation, and attenuated intracellular ROS. Moreover, decellularized ECM promoted the lineage-specific differentiation of BM-MSCs into hepatocyte-like cells, indicated by stronger staining of glycogen, enhanced urea synthesis, and higher expressions of hepatocyte-specific genes.

MSCs have been investigated for an alternative source of cell-based liver regenerative medicine because they have abilities of self-renewal and multilineage differentiation, and there are no ethical issues compared with embryonic stem cells. Sufficient cell number is the primary requirement for cell transplantation because mature hepatocytes have less self-renewal ability. Conventional TCPS monolayer culture system is hard to mimic tissue-specific extracellular microenvironment and results in cell senescence and loss of multipotency of MSCs [21]. Our data showed that cell-deposited ECM successfully accelerated cell growth of BM-MSCs by approximately 4-fold higher than TCPS culture system while maintaining stem cell characteristics, consistent with previous studies [22]. ECM microenvironment also induced the increase and translocation of cyclin D to control cell cycle progression through G_1_ phase to S phase [19]. High level of telomerase activity when cells were exposed to bone marrow-like ECM was possibly responsible for improved cell self-renewal [23]. 

*In vivo*, specific extracellular regulatory microenvironment consists of cytokines, growth factors, and a complex mixture of matrix components to control cell behavior and biological functions of stem cells. Native ECM as an essential part of stem cell microenvironment provides a structural scaffold to resist tensile and compressive stress and functions as a tight connection to cytoskeleton of cells through cell-surface receptors to enable cells to sense and respond to mechanical and chemical signals [24]. To reconstruct liver's stem cell microenvironment we attempted to decellularize native cell-derived ECM while preserving matrix compositions. The fibrillar structure of decellularized ECM was more similar to *in vivo* native ECM than monolayer system [16]. More importantly, decellularized ECM deposited by BM-MSCs were detectable for complicated matrix proteins, including type I collagen, type III collagen, fibronectin, and laminin. However, matrix component of decorin as a small proteoglycan binding to type I collagen fibrils [25] was undetectable after decellularization, suggesting that decorin was soluble and infirmly connected to ECM. The role of decorin in decellularized ECM on cell proliferation and differentiation needs to be elucidated in future.

Accumulation of intracellular ROS, such as superoxide anions and hydrogen peroxide, is thought to cause cell death and inhibit lineage-specific differentiation [26]. Although the mechanisms underlying the influence of ROS on hepatic differentiation of MSCs are poorly understood, increased oxidative stress produced by mitochondria and nicotinamide adenine dinucleotide phosphate oxidase has been demonstrated to induce hepatocyte apoptosis and liver inflammation [27]. It has also been reported that activation of Notch signal pathway protects the survival and biological functions of hepatocytes from ischemia injury by scavenging ROS in mice [28]. Evidence obtained from our present studies showed a significantly lower level of oxidative stress in BM-MSCs cultured on ECM than TCPS. Therefore, it is possible that decellularized ECM promoted hepatic maturation of BM-MSCs by attenuating oxidative stress.

The application of whole decellularized organ is considered as a promising method to reconstruct hepatocyte specific microenvironment and improve the efficiency of MSC transdifferentiation into hepatocyte-like cells [29]. The scarce sources of autologous or allogenic organs and the risk of immunological rejection of xenogenic organs are still obstacles. In addition, various synthetic scaffolds were reported to be used in liver tissue engineering. A collagen-coated poly (lactic-coglycolic acid) (PLGA) scaffold that was fabricated to mimic 3D microenvironment of native liver was shown to support cell survival and increase expressions of liver-specific genes in MSCs [30]. However, the lack of vascular microstructure and simplicity of matrix chemistry remain issues for the design of biomodified scaffolds. To our knowledge, this is the first time to demonstrate that decellularized cell-deposited ECM promoted hepatic maturation from BM-MSCs into hepatocyte-like cells with high expression of hepatocyte-specific genes and increased levels of urea biosynthesis and glycogen storage. The hepatocyte-specific gene expression in TCPS culture system increases during differentiation period; however, long-term culture significantly alters the characteristics of MSCs, evidenced by decreased differentiation potential, high expression of aging genes [31], and shortened telomere length [32]. This jeopardizes the use of MSCs as therapeutic application. Thus, hepatocyte-specific biofunctions of MSCs, that is, improved by ECM culture system in relatively short period will benefit liver tissue engineering.

Type I collagen has been reported to promote hepatic maturation of human pluripotent stem cells [33] and to maintain differentiated hepatocyte phenotypes [34]. In addition, peptides from laminin *α*1 support the biological functions in hepatocytes [35]. In this study, we revealed that cell-deposited ECM was consisted of various matrix proteins that are identical to native liver [29]. Although it is known that various kinds of ECM proteins have an efficiently promotive effect on hepatogenesis of MSCs, the key bioactive component is still unidentified. Meanwhile, ECM derived from different cells supported lineage-specific differentiation, evidenced by opposite influence of osteogenic-specific and adipogenic-specific ECM on controlling differentiation of MSCs into osteoblasts and adipocytes [36] and supportive effect of decellularized ECM derived from synovium MSCs on chondrogenesis instead of osteogenesis [18]. We hypothesize that preservation of the native architecture and complex matrix chemistry provides the mix of structural and chemical signals to drive BM-MSCs differentiation into mature hepatocytes [37]. 

The underlying relationship between BM-MSCs and decellularized ECM was possibly related to mitogen-activated protein kinase (MAPK) signaling cascades. Decellularized ECM suppressed the phosphorylation of focal adhesion kinase (FAK) [38] but induced sustained activation of MAPK and the downstream extracellular signal regulated kinases 1 and 2 (ERK1/2) [19]. Moreover, Xu et al. demonstrated that biosysthesis of bile acid was dependent on the activation of p38 MAPK in primary hepatocytes [39]. In this regard, it is possible that decellularized ECM enhanced the activation of MAPK signaling cascades and thus improved hepatogenesis of BSMCs. However, the underlying mechanism of hepatic differentiation of BM-MSCs on decellularized ECM needs to be elucidated in future studies.

In conclusion, our results indicate that decellularization of cell-deposited ECM preserves the natural framework and matrix proteins. ECM culture system maintains stem cell phenotypes, increases cell proliferative rate, and suppresses oxidative stress in BM-MSCs. We also demonstrate that cell-deposited ECM closely mimics *in vivo* liver's stem cell microenvironment and promotes the differentiation of BM-MSCs to adult liver fates. Our findings therefore contribute to stem-cell-based liver tissue engineering, bioartificial liver development, and clinical stem cell therapies to treat chronic liver damage.

## Figures and Tables

**Table 1:** Primers used for real-time RT-PCR.

Gene	Primer sequence (5′-3′)	GeneBank accession
GAPDH	F: AGAAAAACCTGCCAAATATGATGAC	NM_002046
	R: TGGGTGTCGCTGTTGAAGTC	
CuZn-SOD	F: GGTGGGCCAAAGGATGAAGAG	NM_000454.4
	R: CCACAAGCCAAACGACTTCC	
Mn-SOD	F: GGGGATTGATGTGTGGGAGCACG	BC012423.1
	R: AGACAGGACGTTATCTTGCTGGGA	
ALB	F: TGCTTGAATGTGCTGATGACAGGG	NM_000477.5
	R: AAGGCAAGTCAGCAGGCATCTCATC	
TDO2	F: TCCTCAGGCTATCACTACCTGC	NM_005651.3
	R: ATCTTCGGTATCCAGTGTCGG	
CYP3A4	F: AAGTCGCCTCGAAGATACACA	NM_017460.5
	R: AAGGAGAGAACACTGCTCGTG	
CYP7A1	F: AGAAGCATTGACCCGATGGAT	NM_000780.3
	R: AGCGGTCTTTGAGTTAGAGGA	
CK18	F: AATGGGAGGCATCCAGAACGAGAA	NM_199187.1
	R: GGGCATTGTCCACAGTATTTGCGA	
HNF-4A	F: GGAACATATGGGAACCAACG	NM_178849.2
	R: AACTTCCTGCTTGGTGATGG	

## Abbreviations

MSCs: Mesenchymal stem cells
ECM: Extracellular matrix
BM-MSCs: Bone marrow mesenchymal stem cells
TCPS: Tissue culture polystyrene
ROS: Reactive oxygen species
SOD: Superoxide dismutase
ALB: Albumin
TDO2: Tryptophan 2,3-dioxygenase
CYP7A1: Cytochrome P450 7A1
CYP3A4: Cytochrome P450 3A4
CK18: Cytokeratin 18
HNF-4A: Hepatocyte nuclear factor 4 alpha.
